# Seeing Gaza: Objectivity and Emotion

**DOI:** 10.1007/s11673-024-10362-y

**Published:** 2024-05-22

**Authors:** Genevieve Lloyd

**Affiliations:** University of New South Wales, Sydney, Australia

**Keywords:** Ethics, Objectivity, Emotion, Gaza

If what is unfolding in Gaza is a genocide, it is one that has the novel feature of unfolding under observation in real time across the world. The immediacy of images and live interviews has elicited an answering urgency of emotional response, bringing thousands onto the streets. That is not in itself something new. Images from the Vietnam War had a similar catalyzing effect on activist dissent. Yet, in the context of the daily flow of images from Gaza, it is worth having a close look at the familiar contrast between the objectivity expected of ethical theory and the visceral intensity of engaged sympathy.

Sympathetic emotional response to the distress of others is not peculiar to the human species. Nor is human sympathy directed only to humans; it is readily extended towards other sentient beings, capable of suffering. However, that fellow-feeling is unavoidably subject to limitation. A total absence of empathy—construed as the capacity to imagine oneself into the emotions of others—is understandably regarded as either a pathological deficiency or a character flaw. Yet even normal empathy has its limits and gradations, reflecting an assumed distance—whether literally or metaphorically—from the distress of others. Some degree of emotional detachment is necessary for the continuance of ordinary human living.

The point was eloquently and movingly articulated in the voice of the narrator in George Eliot’s novel *Middlemarch:*

- That element of tragedy which lies in the very fact of frequency, has not yet wrought itself into the coarse emotion of mankind; and perhaps our frames could hardly bear much of it. If we had a keen vision and feeling of all ordinary human life, it would be like hearing the grass grow and the squirrel’s heart beat, and we should die of that roar which lies on the other side of silence. As it is, the quickest of us walk about well wadded with stupidity. (Eliot 1947)

The images from Gaza show ordinary human lives tearing apart under the extraordinary impacts of bombardment. It is the juxtaposition of ordinariness and disintegration which makes them so overwhelming—and yet so compelling. That is why authorities may be unwilling to facilitate access to them; and also why journalists have been willing to die in the interest of making them available.

No picture tells the whole story. Images can be manipulated for political ends, even without the seductive falsifying capacities of Artificial Intelligence. Moreover, exposure to a flood of devastating images may deaden emotional response rather than quickening it. Yet the immediacy of a visual image or live interview, from scenes of catastrophe, foregrounds emotional impact and can intensify emotional response.

Seeing images from Gaza can elicit a jarring oscillation between paralyzed incredulity and impulsive determination to do something—one-knows-not-what—in response. On a common way of thinking, that emotional reaction lies outside the proper domain of ethical theory and deliberation. I want to argue for a different perspective on immediate emotional response, in the context of contemporary global crises. It may seem bizarre to associate the cultivated, professional objectivity of ethical theory with the self-protective detachment which Eliot’s narrator describes as the state of being “well wadded with stupidity.” Yet there are aspects of that idealized objectivity which give rise to some concerns about its own distortions in relation to distance.

Those Gaza images bring distance and closeness together. In some respects, that too is nothing new. Communications technology has accentuated the awareness of global interconnection, reducing the sense of distance. There is, however, something new in increased awareness of the interconnections of global crises under conditions of accelerating climate change—the mass displacements of people; the increased frequency of “natural” disasters related to extreme weather events; the transmission of viruses across borders.

The visibility of distant crises can now serve to unmask the hidden fragility of lives closer by, which are unavoidably caught up in those interconnections. In that context, the reaction of engaged sympathy can become something more than an attitude of altruistic concern for the well-being of distant others—extended from an assumed standpoint of security. It can be experienced more deeply as a shocked realization of shared vulnerability, which challenges habitual separations between “us” and “them.”

How does this shift in emotional response bear on ethical theorizing about contemporary warfare? Much of the human impact of war—now as ever—has to do with emotional devastation: with the grief, the lost sense of future, the shattering of hope, the shared impotent rage. Of course actual experience of emotions is not required in order think theoretically about them; in the grip of deep emotion, it may be impossible to think clearly about anything at all. Yet insight into profound emotional impacts on ordinary human lives is crucial to considering ethical aspects of war.

If the primary focus of ethical theorizing is on abstract principles of action—or on definitional criteria for “war crimes,” “crimes against humanity,” “genocide”—crucial insight into the *upshot* of military strategy or defence policy can go missing. Insight into *upshot* is, in this context, different from the concern with *consequences* which is a familiar theme in some ethical theory. My use of the term “upshot” here is meant to evoke what an action “amounts to”—as distinct from outcomes or consequences, construed as separable from what is actually done. In that respect, finding adequate descriptions of what is done does not always equate with what was intended by the agent; or with a benign description under which they might regard themselves as acting.

Thus, a military engagement might be described by those responsible for it as “self-defence against future attack” or “protecting innocent civilians from terrorists in their midst.” Those descriptions can be accommodated to demands for “consistency with humanitarian rules of engagement” or “compliance with international law.” However, adequate understanding of what is being done in Gaza may demand taking into account also how the military action is experienced by those living there. What is done may amount to the destruction of conditions necessary to life—even if abstract criteria of *intent* for genocide are not satisfied.

The immediacy of those images from Gaza can elicit a shocked realization of what is actually being done. It can render agent-centred descriptions of “self-defence” or “collateral damage” hollow. For the images are focused on those who are subjected to violence, rather than on descriptions under which the violence is perpetrated. It is that immediacy which elicits the observer’s emotional engagement with suffering others; and it is that shift of focus which raises issues of how objectivity should be construed.

Emotional response to the apprehension of suffering in others is commonly regarded as “subjective”—and hence as antithetical to the very idea of “objective” rationality, which is central to ethical theory. I want to suggest that this polarization may involve a distorted way of thinking of objectivity.

Side-lining emotion fits well with a widely endorsed approach to international relations—oddly labelled “political realism.” That approach sees the content of “national interest” in terms of a narrowly construed version of “security” and a broadly construed version of “self-defence”—a gift to the arms industry. A “realist” approach to relations between nation-states has notoriously been associated with the claim that the consideration of international relations has nothing to do with ethics. Of course, not all international relations theory treats ethical considerations as a subjective intrusion into objective thinking. Yet thinking of emotion as antithetical to objectivity does facilitate a tendency within ethical theory itself to treat emotional response as outside its domain.

Underlying binary oppositions between “subjective” emotion and “objective” theorizing, is a long history of the celebration of Reason in Western Philosophy. On a familiar version of that history—associated especially with the Enlightenment—Reason is seen as the defining essence of human beings, distinguishing them from the rest of Nature. In a closely related thread of that familiar narrative, the rightful supremacy of human beings within Nature is reflected in the rightful dominance of Reason within the human mind. Reason is regarded as transcending, dominating, and—where appropriate—suppressing Imagination and Emotion.

That hierarchical model of the human mind is so familiar that it can go unnoticed that the conceptual constructs supporting it are contingent, variably interpreted, and able to be changed. Enlightenment texts themselves offer a range of ways of thinking of the relations between Reason, Imagination, and Emotion. However, on what has become a prevalent reading of the Enlightenment tradition, Reason has been construed in a relation of dominance within the mind. Emotion has then been located, within that hierarchy, mostly in the form of negative “passions” in need of control—if not outright suppression—by Reason.

The celebration of Reason in philosophical texts of the Enlightenment did not indicate a lack of interest in emotions. René Descartes’s *Passions of the Soul,* published in 1649, offered detailed analyses of their general nature and of their interactions. Later in the seventeenth century, Spinoza argued in his *Ethics,* published after his death in 1677, that Reason, Imagination, and Emotion were structurally interconnected. On his account, Reason had the power to “remedy” the destructive force of negative passions only because its exercise involved the mind’s own transitions to greater activity. For Spinoza, that transition involved, by definition, the most powerful of emotions: Joy.

David Hume, mocking a prevailing view of Reason, argued mischievously in his *Treatise of Human Nature*—published in 1739–1740—that, rather than being considered the master of the passions, it should be considered their slave. His contemporary, Adam Smith, in *Theory of the Moral Sentiments,* published in 1759, also challenged the supremacy of Reason by offering a rich account of the empowering relations between imagination and sympathy in the well lived human life.

Philosophical affirmation of the supremacy of Reason became the dominant influence in conceptualizing European ideals of objective thinking. However, the philosophies which offered more integrative ways of thinking of Reason’s relations with Imagination and Emotion offer resources for now re-conceptualizing prevailing models of rational deliberation and theoretical thinking.

Spinoza grounded his treatment of human thought in a distinctive metaphysics of Substance, Mind, and Matter. However, his rich insights into the power of *Affects* in human lives—individually and collectively—stand independently of the intricacies of the whole system. For him the inter-relations of Reason and Emotion yield a dynamic model of theoretical thinking as imbued with emotion, without loss of objectivity.

Hume’s dismissive treatment of the notion of Reason as “master” of the passions unfolds as a treatment of moral judgement as grounded in better understanding the passions rather than repudiating them. Adam Smith’s treatment of emotion in *Theory of the Moral Sentiments* also offers resources for re-conceptualizing prevailing models of Reason. He shows how sympathetic response to the suffering of others is more than a mere passive reverberation. It involves a serious intellectual exercise in imagining oneself into the situation of another.

What is important here about these illustrations is not so much the rival merits of particular philosophical theories. It is rather the fact that the relations between Reason and Emotion have been variously construed and evaluated in the history of philosophy. Those relations can continue to be re-thought in new contexts, revitalizing and enriching—rather than abandoning—old ideals of objectivity.

In the context of contemporary crises, the notion that objectivity demands either disdaining or prescinding from emotion can be seen as a limited—unimaginative—way of thinking of the nature of ethical thought and deliberation. What is at stake here is not the articulation of some novel superior form of cognition. Rather, it is a shedding of misplaced binary oppositions between emotion and theoretical thinking, which can impoverish ethical theory.

Twentieth century English speaking philosophy, especially in the early stages of its preoccupation with “analysis,” tended to model itself on the methodology of Science—construed as a paradigm of objectivity, with capacity to reach definitive understanding of how things really are. Emotion and Imagination were then readily relegated to the realm of the Arts and Humanities. It is an outmoded way of thinking of Science—and, more generally, of theoretical thinking. Yet something of that opposition lingers in largely unexamined assumptions about what is involved in thinking theoretically about ethical issues. It is especially unhelpful in trying to think through the layered complexities of human impacts of contemporary inter-connected global crises.

Watching the unfolding catastrophe in Gaza challenges human capacity to think clearly and coherently in response to the apprehension of horrific situations. It does, however, open possibilities for attempting to think *with* others in those situations—in a solidarity of shared vulnerability, rather than *about* them at an idealized, distorting distance. Theoretical thinking about ethics can be prompted and sustained by emotion—and imbued with its power.

Thinking critically about assumptions in ethical theory may do little now to help the people of Gaza. Perhaps, though, it may in the future help expose the cruelties of what has been done there—whether or not future legal deliberations deem it genocide.
